# The promises of quantitative systems pharmacology modelling for drug development

**DOI:** 10.1016/j.csbj.2016.09.002

**Published:** 2016-09-23

**Authors:** V.R. Knight-Schrijver, V. Chelliah, L. Cucurull-Sanchez, N. Le Novère

**Affiliations:** a Babraham Institute, Babraham Research Campus, Cambridge CB22 3AT, UK; b European Molecular Biology Laboratory, European Bioinformatics Institute (EMBL-EBI), Wellcome Trust Genome Campus, Hinxton, Cambridge CB10 1SD, UK; c GlaxoSmithKline, Gunnels Wood Road, Stevenage, Hertfordshire SG1 2NY, UK

**Keywords:** Quantitative systems pharmacology, QSP, Drug discovery, Modelling, Systems biology, New therapeutic entity

## Abstract

Recent growth in annual new therapeutic entity (NTE) approvals by the U.S. Food and Drug Administration (FDA) suggests a positive trend in current research and development (R&D) output. Prior to this, the cost of each NTE was considered to be rising exponentially, with compound failure occurring mainly in clinical phases. Quantitative systems pharmacology (QSP) modelling, as an additional tool in the drug discovery arsenal, aims to further reduce NTE costs and improve drug development success. Through *in silico* mathematical modelling, QSP can simulate drug activity as perturbations in biological systems and thus understand the fundamental interactions which drive disease pathology, compound pharmacology and patient response. Here we review QSP, pharmacometrics and systems biology models with respect to the diseases covered as well as their clinical relevance and applications. Overall, the majority of modelling focus was aligned with the priority of drug-discovery and clinical trials. However, a few clinically important disease categories, such as *Immune System Diseases* and *Respiratory Tract Diseases*, were poorly covered by computational models. This suggests a possible disconnect between clinical and modelling agendas. As a standard element of the drug discovery pipeline the uptake of QSP might help to increase the efficiency of drug development across all therapeutic indications.

## 1 Introduction

### 1.1 The price of productivity

**Fig. 1:**
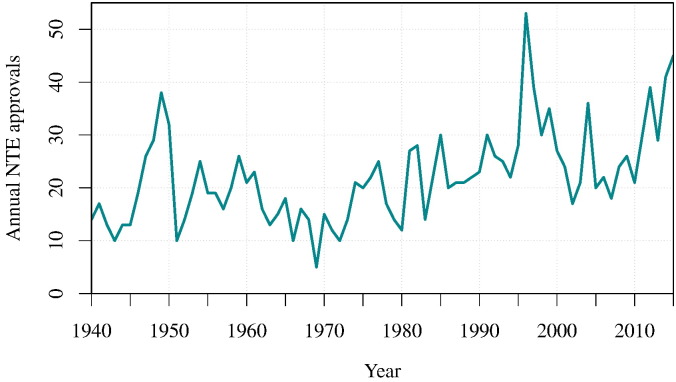
Total new therapeutic entity (NTE) approvals since 1938. New data since 2008 illustrates the recent positive shift in NTE output. The number of NTEs approved in 2014 and 2015 is surpassed only by 1996 when a backlog of new drug applications (NDAs) may have been rapidly processed following a change in regulations. Data was sourced from the Food and Drug Administration (FDA).

**Fig. 2:**
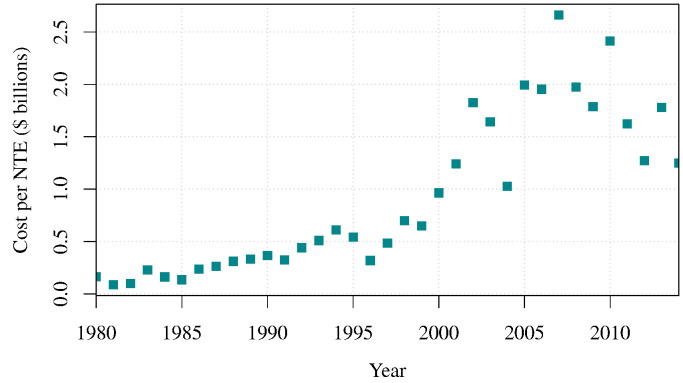
The price of drug development from 1980 to 2014. An exponential increase in new therapeutic entity (NTE) cost is seen before 2008. The cost was calculated using R&D expenditures data given by PhRMA member companies [8] and annual Food and Drug Administration (FDA) reports on NTE approvals seen in Fig. 1. It is assumed that the given PhRMA members' expenditure proportionally represents the global expenditure over time and that these were adjusted for inflation.

At the turn of the 21*st* century, a prevalent view of the pharmaceutical industry productivity was that compound attrition throughout the drug discovery pipeline was increasing [1], [2] and that the annual output of new therapeutic entities (NTEs) was in decline [3]. A broader picture, on the other hand, implies that there had been a tenuous growth in number of annual NTEs approved since 1940 (Fig. 1). NTEs are novel chemical and biological drugs where the active moiety has not previously been approved by the FDA. As a result, they are often used as a measure of pharmaceutical research and development (R&D) output [4]. Despite the apparent decline in NTEs seen over the last two decades, the long-term growth in NTE output appears to be unabated (Fig. 1). The primary concern within the pharmaceutical industry is that dramatic increases were seen in the total cost of bringing each NTE to market [5], [6]; the cost of drug discovery was seen to increase exponentially [7]. However, the evidence suggests here as well that the cost per NTE might have reached a plateau by 2010 following the rise in approvals and may have even been in decline since [8], [9] (Fig. 2). In lieu of this perceived negative trend, there could be instead a positive shift in raw R&D output in the pharmaceutical industry.

The rapidly rising cost of drug discovery may have been, in part, caused by the increasing frequency of compound termination in the highly expensive clinical research phases. Although the cost per NTE may be decreasing (Fig. 2), the contribution of late-stage drug failure to pharmaceutical expenses remains substantial. Drug attrition which occurs during clinical trials stages is caused by unfavourable efficacy, lack of commercial viability and poor safety [10], [11]. To effectively combat this costly termination of drugs, the pharmaceutical industry has been keen to augment the drug discovery process with theoretical and computational modelling [12], [13], [14], [15], [16], [17], [18]. Models offer cheap predictive solutions for drug pharmacokinetics (PK), pharmacodynamics (PD) and patient population responses. Models are also capable of providing novel insights into fundamental biology which furthers our understanding of nature and diseases [19], [20].

### 1.2 Pharmacokinetics, pharmacodynamics and pharmacometrics.

The models by Teorell [21], [22] are often regarded as the foundations of mathematical modelling in pharmacology [23]. PK modelling is largely focused on the absorption, distribution, metabolism and excretion (ADME) properties of compounds, i.e. what the body does to the drugs. It was not until the 1950s that the intrinsic drug activity or pharmacodynamics (PD), i.e. what the drug does to the body, was effectively considered in modelling. To understand and predict the complete effect of drug administration, both elements were combined as PK/PD models [24]. The first dedicated pharmacokinetics software, NONLIN, began distribution in 1969 and signalled the start of a busy period for PK/PD modelling. Multiple advancements in techniques and programs over two decades caught the interest of the FDA who then encouraged the use of quantitative modelling in drug development [25]. At this time, and possibly resulting from this sudden interest, kinetics-mediated drug attrition in clinical phases was dramatically reduced [10]. Traditional pharmacokinetics, pharmacodynamics and statistical pharmacometric models, based on empirical or semi-mechanistic representations, have more recently been complemented by physiologically-based pharmacokinetic (PBPK) models, using more accurate representations of the various body compartments. PBPK models incorporate drug-independent data such as tissue blood flow, which enables inter-compound predictions and inter-species translation [26], [27].

Pharmacometric models are designed to predict the biological variability in patient populations which can be used to predict clinical trial outcomes. Lee et al. studied the success of pharmacological models in FDA approval decisions between 2000 and 2008 [28]. They show a six-fold increase in pharmacometric analyses over the nine years and suggest that the pharmacometric elements were essential for approval in the majority of NDAs [28], [29], [30].The PK/PD success story in preclinical prediction and the continued value of pharmacometrics in clinical stages suggests that more sophisticated and mechanistic pharmacological modelling approaches may help to further reduce the current sources of late-stage attrition.

### 1.3 Mechanistic modelling with systems biology

For the better part of a century, computational modelling and simulation approaches have been essential in understanding biology. Simple simulations of biological systems as early as the giant squid axon model by Hodgkin and Huxley in 1952 [31] illustrate that the integration of such models with experiments can reveal novel emergent properties. The added value of computational models in driving hypotheses has then led to the generation of a separate field of study in its own right, systems biology. Heralded as one solution to understanding the ‘data explosion’ [32], the field has been embraced globally. Systems biology is the interpretation and study of biological networks as a holistic approach. The rationale is that the dynamic response of a whole system, such as the human body, to a stimulus is governed by the collective individual responses of all components of the system. Computational models are then necessary in systems biology because of the complex and multifaceted interactions which can be readily described by mathematical models but do not lend themselves to intuitive understanding.

In discussing the elements of systems biology, Kitano [33] points out that, to fully understand biology at a system-level, one needs to determine the structure of the system. This encompasses the biochemical and physical entities in a network of interactions. Furthermore, the level of understanding also requires knowledge of the time-dependent network interconnectivity. A simple definition has therefore been proposed for systems biology models using the following criteria [34]: A functional model must contain

1. The components that constitute the biological system of interest.
2. The temporal dynamic nature of each individual component of this system.
3. The interconnectivity and temporal dynamic interaction between these components.

Quantitative systems pharmacology (QSP) draws its inspiration from the need for biologically mechanistic modelling, the success of pharmacometrics modelling and systems biology.

### 1.4 Quantitative systems pharmacology

Aiming to incorporate detailed biological processes within PK/PD and PBPK, QSP is the application of systems biology modelling to drug discovery. A working definition was provided initially by Allerheiligen in 2010 [35] and extensively by Sorger et al. in an NIH white paper in 2011 [36]. In brief, QSP is focused on the druggability of targets within biological systems. It relies on dynamic mathematical and computational analyses which integrate multiple experimental data and, similar to systems biology, QSP models are concerned with the emergent properties found in detailed representations of biological systems. Furthermore, the output of QSP is a knowledge-base or model with predictive capabilities to enhance drug discovery. With this summary in mind, an additional criterion can be appended to the systems biology model description [34] to define QSP. A quantitative systems pharmacology model should also include

4. The modulation of components and dynamic interactions by putative therapy or compound(s).

**Fig. 3:**
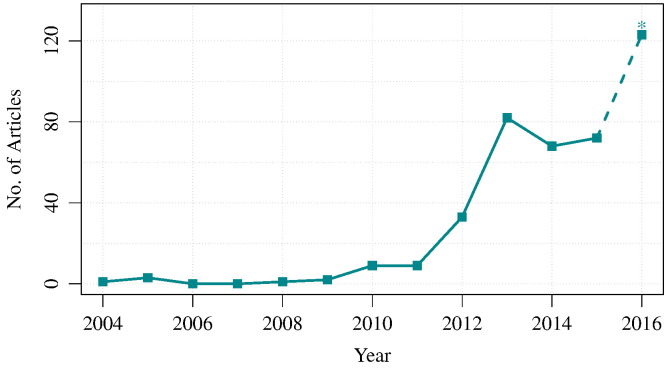
The rise of publications in Systems Pharmacology. A simple search query in Pubmed was used to return all articles explicitly containing “systems pharmacology” in the title, abstract or key words sections (n = 352). Not all abstracts refer to systems pharmacology models. The expected number of articles published in 2016 (*) is a simple prediction based on the number of articles currently available in 2016 (72 × (12/7)). Performed on the 15*th* of July 2016.

In practice, this already appears to be a key aspect of the majority of systems biology analyses. However, this is the principal rationale behind the task of constructing a QSP model. Given the criteria presented here, the construction of a QSP model is reliant upon the existence of quantitative temporal data. This reliance has been a limiting factor for the development and use of mechanistic modelling in drug development in the past. Lately however, the accumulation of relevant data has enabled a reasonable use of QSP models, at least in basic and preclinical research. The match was struck in a white paper published in 2011 [36]. Following this, the quantity of published articles describing systems pharmacology models, as well as reviews of their utility, has grown substantially. As shown in Fig. 3, although the term *systems pharmacology* made a notable appearance in papers from 2010, a 9-fold increase was seen between 2011 and 2013 with 9 to 82 articles published in each year respectively.

This increased interest may indicate that the pharmaceutical industry is beginning to integrate systems-level modelling as a standard element of the drug discovery pipeline. Nonetheless, the mainstream application of QSP modelling and the bringing of modelling into the clinical stages of drug development still requires further effort. QSP modelling has to effectively address the clinical requirements, such as descriptions of statistical patient variability or long-term disease progression, and improve the accessibility of modelling for clinicians and pharmaceutical companies [37]. Further work in perfecting QSP modelling is needed for meeting all expectations. From target identification through to approval, QSP is proposed as the required tool in continuous program evaluation for predicting and minimising the high cost of late-stage drug attrition [35]. How has the mantle been upheld so far? Recent reviews have discussed the potential goals, methods and application of QSP [38], [39], [40], [41]. The present mini-review is a brief attempt to examine the current scope and extent of the QSP modelling field.

## 2 The scope of QSP models

A short examination makes clear that a simple search query, such as that performed in Fig. 3, is not capable of truly retrieving all QSP models in literature. Such an attempt frequently returns reviews and static network analyses but often fails to capture research which can otherwise, given the criteria in 1.3–1.4, be described as systems pharmacology modelling. It is also unrealistic to expect *“systems pharmacology”* or *“QSP”* to appear explicitly in the more dated research abstracts. In fact, we can see that the term *“systems pharmacology”* was not mentioned in Pubmed until 2004 (Fig. 3). Therefore, in an effort to capture the spread of QSP modelling across therapeutic areas and indications, we constructed a more complex, wider and more precise, text mining query to recall relevant research abstracts from Medline dated from 1965 to 2015. The query included a large set of positive key terms including, amongst others, *systems pharmacology* and *mathematical model* (The full text mining analysis will be presented in a more detailed manner in a separate research paper). Initially, a set of negative terms were also included. However these substantially decreased the recall of the positive control and reduced the total number of modelling abstracts retrieved. The abstracts and clinical studies for this time period were annotated with Medical Subject Headings (MeSH) 2014 and categorised into the separate disease branches.

**Fig. 4:**
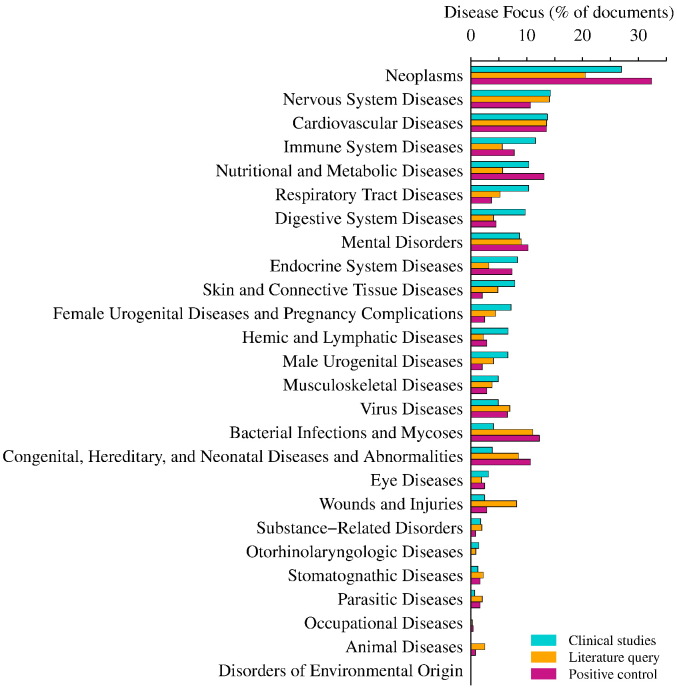
Clinical trials and modelling. Clinical studies, modelling literature and positive control abstracts were labelled with diseases by their in-text occurrence. Each disease was categorised under MeSH 2014 disease branches and documents without any disease were omitted. The fraction of documents labelled with each disease was calculated using the *n*_*d*_ for each corpora (Table 1). The software I2E^©^ 4.2 (Linguamatics) was used to run the query and perform the MeSH term extraction.

The result of this analysis shows that while generally matching the agenda of clinical trials, in a few disease categories, the focus of modelling literature is poorly aligned with clinical interest. For example, Fig. 4 shows that the fractions of documents discussing *Immune System Diseases*, *Respiratory Tract Diseases* and *Digestive System Diseases* were approximately two-fold greater in clinical studies than in the modelling literature. Therefore, certain diseases may lack an extensive modelling arsenal. Conversely, categories such as *Bacterial Infections and Mycoses* or *Congenital, Hereditary, and Neonatal Diseases and Abnormalities* were commonplace in modelling literature but were comparatively much less prevalent in clinical trials. Unsurprisingly, there appeared to be little clinical interest in *Animal Diseases* (Fig. 4).

The BioModels database was included in this assessment as a positive control for dynamic biological models since it is the largest public repository of curated mathematical models in systems biology [42], [43]. The query retrieved 76% of all abstracts with models stored in BioModels. However, only 35% of the models stored in BioModels were labelled with a disease term (Table 1). Note that a given document could be tagged for multiple disease categories. One caveat of this data set is that it may not be enriched for criterion 4 in defining a QSP model. Whereas the image resulting from the text mining analysis may not portray the full scope of QSP or pharmacological modelling, the fraction of positive control articles retrieved suggests that the recall of models by the query was reasonable. However, samples indicate that, whilst 18% of total Medline abstracts were systems modelling papers, only 5% of the total abstracts retrieved were pharmacological models.

The current approach made no distinction between QSP and PK/PD or pharmacometrics models and, as the majority of retrieved documents were not systems biology or pharmacology, a poor overall precision was expected. Since this study only considered abstracts available in Medline, it did not take into account unpublished in-house modelling works and published materials not indexed in Medline. Moreover, explicit textual references to a disease were required for a document to be categorised within MeSH disease branches. Therefore, high-resolution models that mentioned specific genes, proteins or pathways without explicitly mentioning the disease were missed. Finally, other errors may lie within the classification itself. The MeSH branch *Pathological Conditions, Signs and Symptoms* was omitted due to its ambiguous nature. Annotation of MeSH terms can be inconsistent or false and some diseases, often tissue-specific cancers, fall into multiple categories. Clearly the current analysis can be improved on the side of precision.

All that said, with a few exceptions, the spread of diseases covered by BioModels broadly spanned the spectrum of models mentioned in Medline. A concerted effort within the BioModels database has focused on models of diabetes [44]. This may explain why the focus of *Nutritional and Metabolic Diseases* or *Endocrine System Diseases* in clinical studies is closer to that of the BioModels database than the overall modelling literature (Fig. 4).

## 3 The extent of QSP models

The consistent application of computational modelling analyses in NDA success is clear evidence for the beneficial application of theoretical models in drug discovery [29]. QSP models also provide a method to further understand the biological mechanisms and predict the ideal targets, drug dose and toxicological properties. This improves the confidence in drug efficacy and safety profiles prior to success of an NDA. One particular and compelling use case of QSP modelling is the application of a calcium homeostasis model by Peterson and Riggs [45][BioModels Database: MODEL1604270004]. Khurana et al. adapted a publicly accessible version of the model specifically for the FDA review of a Biologics License Application in 2014 [46]. This model was then used to explore the kinetics and drug administration of a recombinant human parathyroid hormone. Simulations demonstrated that the dose regimens or drug properties could be optimised to provide a better adherence to the therapeutic window. In fact, a clinical study has been filed this year following the conclusions drawn from the QSP dose optimisation.

Below, we present several other example cases illustrating the application and utility of QSP models in diseases of clinical priority.

### 3.1 Neoplasms

Neoplasms were labelled in 27% of clinical studies, confirming that oncology is highest on the clinical agenda (Fig. 4). QSP has analysed the spatio-temporal dynamics of tumours with models of tumour growth, drug delivery and intervention efficacy [47], [48]. For instance, the QSP model by Sharan and Woo [49] [BioModels: MODEL1604270003] integrated aspects of growth, signalling and biomarkers. The model explored treatment with the anti-vascular endothelial growth factor (VEGF) agent sunitinib. A further modelling exercise combined the mechanistic signalling module with additional clinical responses [50]. This highlighted the benefits of effective biomarker monitoring and dose optimisation in complex cancer therapy. Preclinical analyses of drug combinations is one application of modelling efforts in cancer. A model by Zhu et al. [51] [BioModels: MODEL1604270000, MODEL1604270001] assessed the dose patterns of two anti-pancreatic cancer molecules, gemcitabine and birinapant in cell culture. Although the model was not further translated to an organismal scale, it illustrated how preclinical modelling can shortlist optimal dose schedules for clinical trials in contrast to current regimens [52].

### 3.2 Nervous system diseases

Drug development in central nervous system (CNS) diseases such as Alzheimer's disease has been plagued by poor translation of efficacy from animal models into humans [53], [54]. Low predictive precision in animal models is often caused by significant differences in neuronal circuitry between species as well as unknown CNS biology and disease pathology. Using mathematical interpretations of the known processes, systems modelling is capable of testing multiple hypotheses to predict the possible mechanisms of drug action. The quantifiable nature of this approach is also ideal as a translational step or compliment to animal models [55]. For example, by humanising parameters derived from animal models in a dynamic network, it may be possible to predict the outcome of an intervention in humans before clinical trial commencement. This further be used to specify the compound properties, if any, required for drug success in humans where a compound may not appear to be efficacious. Low patient efficacy in clinical trials of drugs for CNS diseases means that early indication of clinical effect, or lack thereof, is highly important in decision making. For instance, Nicholas et al. [56] developed a QSP model to evaluate the potential of 5-HT_4_ receptors as a target to treat Alzheimer's disease. They coupled a simple cortical neuron model with a ligand competition module for 5-HT_4_ receptor binding. The model demonstrated that low efficacy 5-HT_4_R agonists would worsen the disease state when assessing the clinical outcome of a serotonergic agonist in scopolamine-induced cognitive deficit patients. It was shown that a threshold intrinsic activity of the agonist-receptor complex was necessary to modulate the pathway for improvements on patient cognition.

Roberts et al. [57] developed a similarly structured model with the NEURON software and used a QSP approach to study the attenuation of parkinsonian hypokinetic motor symptoms. The model integrates the neuronal circuitry of the cortico-striatal-thalamic loop with ligand-receptor competition dynamics. The local field potential of the subthalamic nuclei was used as a clinical marker. Calibrated using the polypharmacology of 43 anti-psychotic drug combinations, the clinical outcomes of dopamine, NMDA, adenosine, 5-HT modulators and even placebo treatments were predicted in simulations. This method quantified which targets could generate the optimal response and which compounds offered the greatest therapeutic potential. When directly parametrised with human data, simple neuronal circuitry models mitigate the problems arising from species translation.

### 3.3 Cardiovascular diseases

Mechanistic models have greatly contributed to the fundamental understanding of cardiovascular diseases from signalling [58] to fluid dynamics [59]. In therapeutics, models of drug-eluting arterial stents have been used to explore the mechanisms and increase efficiency of stent development [60]. Garmaroudi et al. [61] presented a combinatorial approach to pathway perturbation in cardiovascular disease signalling. They used QSP modelling to evaluate options of polypharmacology in minimising adverse reactions and countering biological redundancy; systems modelling enabled researchers to examine the network-wide effects of multi-target perturbations. The model assessed the ability of several compounds to restore impaired nitric oxide-cyclic guanosine 3*′*,5*′*-monophosphate (NO^⋅^-cGMP) signalling. The authors simulated the dynamic elevation of cGMP resulting from 377 different individual, double or triple parameter perturbation combinations. One optimal triple reaction perturbation was predicted to increase cGMP substantially beyond all other combinations. The predictions were then validated *in vitro* using clinically available compounds.

Cardiotoxicity is associated with cardiovascular diseases, and an important hurdle in drug discovery. Individual drugs and drug–drug interactions can stimulate cardiovascular arrhythmia as an adverse reaction. Computational modelling efforts have been launched to define the mechanisms underlying the effects of a multitude of compounds on arrhythmic events as well as their therapeutic windows for a multitude of compounds [62]. In forming QSP models, the *in silico* reconstruction of known cardiac biology could be combined with the observed clinical outcomes to predict drug-mediated cardiovascular diseases. For example, a PBPK model combined with a response model of cardiomyocyte ion-channels [63] was used to predict the drug–drug interaction of domperidone and ketoconazole on QT prolongation. These cardiovascular disease examples demonstrate that QSP modelling can explore the promising prospects of polypharmacy in drug-discovery [64].

### 3.4 Nutritional and metabolic diseases

The global incidence of nutritional and metabolic diseases such as diabetes is steadily rising. Clinical efforts to understand and treat diabetes have been mirrored by a large plethora of models comprehensively summarised by Ajmera et al. [44]. The scales of these models range from intracellular signalling to systemic homeostasis and disease progression. One recent QSP model explored the current assumptions underlying the therapeutic mechanism of interleukin-1*β* (IL-1*β*) blockade in type-2 diabetes mellitus (T2DM) [65] [BioModels: MODEL1604 270002]. The authors described a QSP disease progression model of T2DM including a model of *β*-cell function. The simulation of therapeutic perturbations explored the facets of anakinra treatment. The model predicted that improved *β*-cell function is responsible for its short-term efficacy. Additionally, predictions estimated that a persistent improvement in disease state was governed by an increased *β*-cell mass but only after sustained therapy. They concluded that (IL-1*β*) suppression over several years may be required to reach a significant therapeutic endpoint.

### 3.5 Diseases with nascent QSP

Contrary to cancer, CNS and cardiovascular diseases, several disease categories have rarely been studied using computational modelling. However, the situation is changing in a few cases.

A recent platform was designed to simulate the systemic immune response to pathological disruptions. The fully-integrated immune response model (FIRM) describes the effects of several cytokines upon a variety of cell types [66] [BioModels: BIOMD0000000608]. The application of FIRM was demonstrated on tuberculosis, tumour rejection and pathogen response. Adoption of FIRM as a QSP environment could prove useful in predicting the risk of compromising the immune system by compounds with immunosuppressive properties.

Substantial clinical interest in respiratory tract diseases has encouraged the construction of a QSP model for exploring the mechanisms underlying the effects of a 5-lipoxygenase inhibitor, Zileuton, on asthma [67] [BioModels: BIOMD0000000490]. The model explores target comparison, proposes an emergent explanation for clinical data and discusses optimal dose strategies.

In the gastrointestinal system, modelling the interaction between gut and drugs is obviously key to optimising drug oral administration. However, only a very small fraction of QSP research focuses on diseases of the digestive systems (note that we are only talking about QSP models, agent-based models typically use the gut epithelium as the tissue of choice [68]). Modelling intestinal *C. difficile* infection, Leber et al. generated a model exploring the immune response of the gut [69] [BioModels: BIOMD0000000583]. The model predicts that suppression of the gut immune response may, counter-intuitively, aid recovery from infection and perhaps assist in fecal transplant therapy. Expansion of QSP studies into sparsely modelled (but otherwise clinically important) diseases may provide ways to reduce late-stage attrition. The ease at which modelling can be performed will be crucial to achieve this expansion.

## 4 Modelling is easy these days

Common modelling approaches used in systems biology can be easily applied to QSP, and many tools are available for the curious researcher [70], [71]. For example, data obtained from open-access databases of biological networks such as KEGG Pathways [72] or Reactome [73] can be used with programs like Cytoscape [74] and CellDesigner [75]. To describe a dynamic system, mathematical equations can be defined and solved in a wide variety of freely available environments such as Copasi [76], R [77] or Octave [78]. Proprietary environments such as SimBiology^®^ (part of MATLAB^^®^^[79]) are also often used for model development. Finally, some commercial drug development programs specialise in predicting the ADME properties of compounds, like Simcyp [80] or Gastroplus™.

A typical modelling work flow relies on several software tools and the communication between tasks or between researchers must be seamless. As mentioned by Leil and Ermakov [81], the lack of acceptable standards and tools could hinder the emergence and commonplace use of QSP in drug discovery. Standardisation efforts facilitate model sharing, reproducibility of their analysis and ultimately domain repute and progression. In systems biology the Systems Biology Markup Language (SBML) [82] has been a *de facto* standard for more than a decade. Developed by the community, it is supported by hundreds of software tools, databases and modelling platforms. The pharmacometrics community recently developed the Pharmacometrics Markup Language (PharmML) [83]. PharmML was developed through the DDMoRe consortium [84], a partnership between academic partners and the pharmaceutical industry. It was designed as a common format or *lingua franca* between a large variety of popular pharmacometrics tools and aims at establishing a standard language for PK/PD and QSP modelling. Common formats such as PharmML and SBML encourage the sharing of models, fuelling scientific collaboration. This also promotes model progression, reuse and much needed training of new translational scientists in both academia and industry.

The DDMoRe project also aims to create and improve open software resources available for modelling in drug development. For instance, it provides a freely available repository for sharing pharmacological models (http://repository.ddmore.eu/). BioModels [42] itself provides an increasing number of curated pharmacological models, including PK/PD models (the models mentioned in this review are freely available in BioModels). Placing models in open-access repositories increases their visibility and the availability of their code allows them to be reused. This is of great importance as it may help to concentrate computational efforts and provide clinically relevant models to researchers.

## 5 Summary and outlook

The number of systems pharmacology articles published has increased dramatically since 2011 bringing an array of quantitative models (Fig. 3). These models are capable of capturing dynamic systems at varying scales, simulating the purported effect of drugs and strategies used in clinic, and assessing the validity of our current biological understanding and clinical outcomes. Here we have briefly discussed examples of the application and extent of current QSP modelling, in particular for disease categories rich with models. A preliminary evaluation of the disease focus suggests that the efforts of modelling could be align better with the clinic.

Both *Cardiovascular diseases* and *Nervous systems diseases* are prone to frequent compound failure [12]. In these complex disease categories, high drug attrition rates may partially explain the popularity of seeking a modelling approach. A traditionally large modelling effort is, however, not the assurance of success. While *Neoplasms* is the most popular disease category for modelling, oncology has the highest compound failure rate in phase II and III trials [10], [12], [85]. An explanation could be the large number of post-mortem models, built following terminated clinical trials. Whilst these are useful in both model validation and for identifying the causes of termination, failure of the compounds may have been foreseen by the earlier use of modelling.

In a follow-up review on the FDA's application of the calcium homeostasis model [45], [46], Peterson and Riggs suggested that the QSP modelling field will be emboldened by milestone cases such as the Natpara^®^ BLA in 2014 [39]. However, they also stressed that several regulatory factors impede a sudden embrace of QSP models in the pharmaceutical domain. Notably, the proper education and training of future QSP scientists is an important prerequisite for industrial interest, as discussed in detail by Sorger et al. [36]. Paramount however, and governing the rate at which QSP is adopted, is the frequency at which key models are successfully put to the test. The accumulation of positive use cases may encourage a shift towards the much needed earlier applications of modelling in the drug development pipeline.

Community-wide interest in QSP is nevertheless increasing. Following the two NIH QSP workshops in 2008 and 2010, reviews have been published annually which offer in-depth discussions on the range of applications, examples and insights offered by QSP modelling [38], [40], [81], [86], [87], [88], [89]. A UK Quantitative Systems Pharmacology Network now funded by the EPSRC aims to bring together experimental and theoretical scientists in both academia and industry. Within the QSP community it is clear that QSP is not just aimed at developing single-use models, but that it is a central and core component of the drug development pipeline. The real worth of QSP is perhaps in integrating the multiple aspects of a compound's development, incorporating the fundamental disease biology, pharmacokinetics and pharmacodynamics including toxicity and clinical outcomes. QSP models could act as central knowledge repositories, improving data communications between collaborating teams. Such a radical pipeline reshuffle may be key to refreshing the drug discovery paradigm.

It is heartening to note the recent positive trend in annual NTE output. Partial responsibility may lie in the increased application of pharmacometrics in NDA approvals [28] with 80–90% of drugs being successful in the FDA review process [7], [12]. However, the fraction of drugs that make it through clinical trials to the NDA stage is only 12% which contributes to the substantial cost of pharmaceutical R&D [12]. QSP can address the efficacy and toxicity which causes this attrition by exploring the pathological mechanisms underlying the disease. The models provided by QSP can be used to document a drug's likelihood of approval which can inform decision making throughout the preclinical and clinical stages. Therefore, instead of resorting to post-mortem analysis, the early and preventative incorporation of QSP modelling into the drug discovery process can increase R&D efficiency by regularly evaluating a compound's viability.

Just as PK models already drove down costly attrition, QSP models, as a predictive platform, may further reduce late-stage drug failure in tomorrow's drug discovery pipeline, the impact of which can only be positive for industry and patients alike.

## Funding

Research was funded by a Biotechnology and Biosciences Research Council (BBSRC) industrial CASE studentship in collaboration between the Babraham Institute and GlaxoSmithKline Research & Development Ltd (NLN, BBSRC Institute Strategic Programme [BBS/E/B/000C0419]; VKS, BBSRC-GSK CASE studentship [BB/L502224/1]).

## Figures and Tables

**Table 1:** The three corpora used in this analysis. Due to technical limitations, fewer documents were labelled with diseases (*n*_*d*_) than the total number of documents in each corpus (*n*_*t*_).

Corpus	*n*_*t*_	*n*_*d*_
Clinical studies^a^	177,609	147,235
Modelling literature^b^	215,097	85,676
Positive control literature^c^	687	244

${}^{n_{t}}$ total number of documents; ${}^{n_{d}}$ number of documents labelled with a disease.

a clinicaltrials.gov.

b Medline — text mining query for models.

c BioModels database.
